# Dietary intake and lifestyle practices of eastern mediterranean postpartum women before and during COVID-19 pandemic: An internet-based cross-sectional survey

**DOI:** 10.3389/fnut.2022.932418

**Published:** 2022-08-04

**Authors:** Reema Tayyem, Nahla Al-Bayyari, Narmeen Al-Awwad, Haya Abuhijleh, Reem Hoteit, Radwan Qasrawi, Eman Badran, Asma Basha, Sabika Allehdan, Khlood Boukari, Jamila Arrish, Rania Abu Seir, Maha Hoteit

**Affiliations:** ^1^Department of Human Nutrition, College of Health Sciences, QU-Health, Qatar University, Doha, Qatar; ^2^School of Agriculture, The University of Jordan, Amman, Jordan; ^3^Department of Nutrition and Food Technology, Faculty of Al-Huson University College, Al-Balqa Applied University, Al-Salt, Jordan; ^4^Department of Clinical Nutrition and Dietetics, Faculty of Allied Health Sciences, The Hashemite University, Zarqa, Jordan; ^5^Hariri School of Nursing, American University of Beirut, Beirut, Lebanon; ^6^Department of Computer Science, Al-Quds University, Jerusalem, Palestine; ^7^Department of Computer Engineering, Istinye University, Istanbul, Turkey; ^8^School of Medicine, The University of Jordan, Amman, Jordan; ^9^Department of Biology, College of Science, University of Bahrain, Bahrain; ^10^Department of Clinical Nutrition, Faculty of Applied Medical Sciences, Taibah University, Medina, Saudi Arabia; ^11^National Nutrition Committee (NNC), Saudi Food and Drug Authority (Saudi FDA), Riyadh, Saudi Arabia; ^12^Department of Biomedical Technology, Al-Quds University, Jerusalem, Palestine; ^13^Faculty of Public Health, Lebanese University, Beirut, Lebanon; ^14^PHENOL Research Group (Public Health Nutrition program-Lebanon), Faculty of Public Health, Lebanese University, Beirut, Lebanon

**Keywords:** postpartum women, COVID-19, maternal nutrition, dietary intake, USDA recommendations, adherence

## Abstract

**Background:**

During the lockdown period, a substantial group of these women reported lifestyle changes.

**Aim:**

The aim of the study is to characterize the dietary patterns, intake and the adherence to the United States Department of Agriculture (USDA) pregnancy guidelines before and during the COVID-19 pandemic in Eastern Mediterranean postartum women.

**Methods:**

An internet-based cross-sectional survey was used to collect the data. The survey was carried out among 1,939 postpartum women from five countries from the Eastern Mediterranean region. Change in dietary intake from the five food groups and the adherence to USDA's daily recommendations were assessed.

**Findings:**

There was a significant increase in the mean (SD) consumption of all the food groups, including bread, rice, and other cereals, fruits, vegetables, milk and milk products, white and red meat, and nuts during the pandemic. Around 84% of participants reported no/low adherence (0–2) to USDA guidelines, whereas only 15% reported moderate or high adherence (3–5) to the guidelines before the pandemic. However, there was an increase in the proportion of subjects reporting moderate/high adherence (22%) during the pandemic.

**Discussion and conclusions:**

A substantial proportion of our study participants reported a lower dietary intake than the recommended amounts, and low adherence to the five food groups. Reasonable and applicable actions should be taken to protect postpartum women and their children from the effects of low dietary intake, particularly during pandemics and lockdowns. More researches are needed to identify the modifiable factors which could improve the nutritional status of the postpartum women during the pandemic.

## Problem or issue

Limited evidence exists about the dietary patterns, intake and the adherence to the USDA pregnancy guidelines before and during the COVID-19 pandemic in Eastern Mediterranean postpartum women.

## What is already known

No studies available investigated the dietary patterns and the adherence to the USDA pregnancy guidelines before and during the COVID-19 pandemic in Eastern Mediterranean postartum women.

## What this paper adds

A considerable percentage of the study participants reported a lower dietary intake than the recommended amounts, and low adherence to the five food groups. More researches are needed to identify the modifiable factors which could improve the nutritional status of the postpartum women during the pandemic.

## Introduction

The postpartum period is a physiological stress period characterized by significant metabolic and hormonal changes (1). During this period, mothers are under a lot of stress and worry about how they will regain their health and shape after childbirth, which can lead to psychological concerns or disorders in certain cases (1). When compared to the attention given during pregnancy, mothers' and their newborns' health during the postpartum period has been neglected in both developed and developing countries (2). van der Pligt et al. (3) stated that postpartum women received less advices about healthy diet and physical activity as compared to during pregnancy (3). It is well-known that food habits and practices vary among people and are influenced by their culture. Whether their food supplies are limited or abundant, women's eating habits are influenced by their socio-cultural context. These factors will have an impact on their postpartum eating habits (2).

A healthy dietary pattern, which is characterized by fruits, vegetables, whole grains, and fish, may decrease the risk of many diseases, and cannot be overstated (1). Adequate nutrition is critical throughout the months after childbirth because the mother should recover after delivery and any nutrients ingested by the mother pass to the infant through breast milk (4). On the other hand, inadequate nutrition during the postpartum period has been associated with the development of a variety of illnesses; iron deficiency, for example, is a primary cause of chronic anemia. Iron deficiency anemia was discovered in the early postpartum period and was found to exist in 21% of women 1 month postpartum (4).

The majority of postpartum women in different countries do not receive any nutritional advice since individual dietary assessment is not a part of the hospital stay after childbirth or normal medical visits during the postpartum period (4). Nonetheless, postpartum care is very important and must include the prevention, early detection, and treatment of complications and diseases. Therefore, counseling and assistance on breastfeeding, birth spacing, immunization, and maternal nutrition must be included during postpartum care (2, 4).

Following the initial outbreak of the disease caused by the SARS-CoV-2 virus in Wuhan (China) in December 2019, the WHO declared a global health emergency, and the disease was quickly classified as a pandemic (5). There is a limited amount of information on how the novel coronavirus affects postpartum women. In addition to that, women may experience increased risks as a result of COVID-19, which might affect their diet, nutrition_habits, and access to nutrition services (6, 7). The impact of the COVID-19 pandemic is expected to be context-specific and differ depending on a variety of country-specific factors (8). It was reported that poor dietary quality was associated with postpartum depression in Chinese lactating women, where depressed women tended to have a more inadequate intake of vegetables and a more insufficient variety of foods (9). A significant decrease was observed in the consumption of fruits, followed by vegetables, and then salted and sweet cereals from pregnancy to post-partum in Spain. Besides that, a decreasing consumption of healthy food from the first trimester to the post-partum period was also observed (10).

The aim of the study is to characterize the dietary intake of the major food groups and the adherence to the USDA's dietary guidelines before and during the COVID-19 pandemic in Eastern Mediterranean postpartum women.

## Methods

### Study design and setting

In this cross-sectional observational study, a web-based questionnaire was used to compare the food intake of women during the COVID-19 pandemic to the five food groups recommended by the United States Department of Agriculture (USDA) from July-December, 2020 (11). Majority of the questionnaire elements were verified during a study conducted on postpartum women in 2018 (more than 200 pregnant women) (12, 13). There were five sections in this survey, which include sociodemographic characteristics, maternal medical history, eating patterns and food frequency questionnaire, physical activity level, anthropometric data, smoking, mental health, and food-related behaviors. The questionnaire's first section included questions about pregnancy and its progression, such as the age of the mother, health status, comorbidities, and other socio-demographic related questions including education, residency, and economic situation. The second section of the questionnaire included questions about the daily serving sizes of common food (bread, pasta, cereal, vegetables, fruits, meat, poultry, fish, and nuts) and the frequency of their consumption preceding completion of the survey. Other questions in the survey covered women's habits prior to COVID-19 pandemic. These questions asked about their mental health, smoking status, and physical activity level. The questionnaire included multiple-choice questions and other questions with open-ended answers. Each participant was informed about the study goal and assured of the confidentiality of their information before completing the questionnaire. The questionnaire was distributed through email, along with links to the website where subjects in the study could complete it. The questionnaire was completed voluntarily and anonymously, and participants had to consent to take part in the study.

### Variables and measurements

#### Body mass index

Postpartum body mass index (BMI) categories were recorded for each participant as per the World Health Organization (WHO) classification (14). BMI categories include underweight, normal, overweight, obese class I, obese class II, and obese class III.

#### Depression

To assess depression in postpartum women, the validated Patient Health Questionnaire (PHQ-9) was used (15). The questionnaire covered nine topics related to depression and had a score range of 0–3, with 0 being never and 3 almost every day. In this study, depression levels were classified as follows: no depression = 0–4, mild depression = 5–9, moderate depression = 10–14, moderately severe depression = 15–19, and severe depression ≥20. PHQ-9 was used in this study because it has been shown to be an efficient tool for detecting depression.

#### Anxiety

Anxiety symptoms were assessed using the clinically validated seven-item Generalized Anxiety Disorder-7 (GAD-7) (16). Participants responded to the GAD-7 survey by ranking items on a four-point scale ranging from 0 (never) to 3 (almost every day). Total anxiety severity was determined by scores ranging from 0 to 4 for no anxiety, 5–9 for mild anxiety, 10–14 for moderate anxiety, and ≥15 for severe anxiety.

#### Physical activity

Levels of physical activity were self-reported by the study participants. Physical activity levels were recorded as low, moderate, or high for any postpartum woman that participated in at least half an hour of daily physical activity (17).

#### Dietary guideline for postpartum women

The classification of recommended food amounts was made using the USDA guidelines for postpartum women (18). Two categories of food groups were developed using the USDA guideline cutoff points, and a score of 0 or 1 was provided for each food group based on the UDSA daily recommendations, with a score of 0 indicating lower intake and a score of 1 indicating higher intake.

The first food group is *Bread, rice, and other cereals*, and the score was provided for lower intake when serving is <6, or higher intake when serving is more than or equal to 6. The second food group is *Fruits*, and a lower intake of fruit is 2 servings, where as a higher intake is more than or equal to 2 servings. *Vegetables* represent the third food group; <2.5 servings indicate a lower intake, while 2.5 or more servings indicate a higher intake. Another food group is *Proteins*, where servings of <5.5 indicate a lower intake, and more than or equal to 5.5 servings indicate a higher intake. The last food group is *Dairy*, a lower intake is <3 servings, while more than or equal to 3 servings indicate a higher intake. Moreover, adherence to each food group was tracked and recorded in this study, and the score of adherance was added to each individual food group. The variable was then divided into two categories to indicate low adherance or high adherance. Low adherance is represented by a score between 0 and 2, and high adherance is represented by a score between 3 and 5.

### Inclusion/exclusion criteria

Participants were chosen at conveniently, and data collection was carried out under certain criteria, which include: (i) postpartum since the pre-COVID-19 pandemic period; (ii) pregnancy of normal course; (iii) age >18; (iv) place of residence is in the five countries listed; (v) responding to all questions; and (vi) participants providing written consent to participate in the study. However, conception during the intra-COVID-19 pandemic period, as well as specific risk factors such as miscarriage, were exclusion criteria.

### Statistics and data analysis

Study factors such as sociodemographic characteristics, health status, and other study cofactors were summarized as descriptive statistics. Means and standard deviations (SD) were used to express continuous variables. Frequencies and percentages were used to represent categorical variables. The Chi-square test was applied to verify any statistically significant difference between the five countries involved in this study, and each categorical variable. The ANOVA test was applied for continuous variables. To compare continuous variables before and during the pandemic, the paired sample *t*-test was used, while the McNemar test (a marginal homogeneity test for paired data) was used to compare categorical variables. The statistical significance level was set at *p* < 0.05, and the statistical analysis was performed using SPSS version 25.

## Results

### Sociodemographic characteristics

A total of 1,630 postpartum women completed the questionnaire. Table 1 shows that the mean age (SD) of the study participants was 28.9 (5.1) years, with Bahrain having the highest mean age of 29.9 (5.5) years and Palestine having the lowest mean age of 27.7 (4.9). Moreover, most of the study participants were from Lebanon (*n* = 604) and the overall study subjects belonged to the young adults age subgroup, as 79.5% were older than 25 years, with Jordan having the highest proportion (84.8%) and Palestine having the lowest proportion (74.4%) of this age subgroup (*p*-value < 0.001). More than half of the study participants own a bachelor's degree (53%), with Saudi Arabia having the highest proportion (72%) and Lebanon having the lowest (36%) among all. On the other hand, the highest proportion of participants having a graduate degree is in Lebanon (27%; *p*-value < 0.001). The majority of the women in this study are unemployed (60.4%) with Palestine having the highest percentage of unemployed women (67%), while Bahrain, on the other hand, has the highest percentage of employed women (48.9%; *p*-value < 0.001). Only a few subjects reported an increase in the family income (6.1%), whereas the vast majority reported a drop in their family household income (57.1%), with Jordan having the highest percentage of this drop in income (75.3%) and Bahrain having the least drop (27.3%; *p*-value < 0.001).

**Table 1:** Sociodemographic characteristics of the study postpartum women (*n* = 1,630).

**Variable**	**All** ***n* = 1,630**	**Jordan** ***n* = 223**	**Palestine** ***n* = 391**	**Lebanon** ***n* = 604**	**Saudi** **Arabia *n* = 324**	**Bahrain** ***n* = 88**	***p*-Value**
**Mean (SD)**							
**Age (year)**	28.9 (5.1)	29.5 (5.1)	27.7 (4.9)	28.8 (4.7)	29.7 (5.4)	29.9 (5.5)	0.020
***n*** **(%)**							
Youth (18–24)	334 (20.5)	34 (15.2)	100 (25.6)	125 (20.7)	61 (18.8)	14 (15.9)	<0.001
Young adults(>25)	1,296 (79.5)	189 (84.8)	291 (74.4)	479 (79.3)	263 (81.2)	74 (84.1)	
**Education level**							
Less than high school	77 (4.7)	26 (11.7)	13 (3.3)	27 (4.5)	9 (2.8)	2 (2.3)	<0.001
High school diploma	207 (12.7)	50 (22.4)	61 (15.6)	60 (9.9)	17 (5.2)	19 (21.6)	
Diploma	222 (13.6)	23 (10.3)	37 (9.5)	131 (21.7)	21 (6.5)	10 (11.4)	
Bachelor's degree	863 (52.9)	100 (44.8)	259 (66.2)	219 (36.3)	233 (71.9)	52 (59.1)	
Graduate degree	261 (16.0)	24 (10.8)	21 (5.4)	167 (27.6)	44 (13.6)	5 (5.7)	
**Employment status**							
Employed	646 (39.6)	91 (40.8)	129 (33.0)	271 (44.9)	112 (34.6)	43 (48.9)	<0.001
Unemployed	984 (60.4)	132 (59.2)	262 (67.0)	333 (55.1)	212 (65.4)	45 (51.1)	
**Family income**							
Decreased	930 (57.1)	168 (75.3)	279 (71.4)	329 (54.5)	130 (40.1)	24 (27.3)	<0.001
Increased	100 (6.1)	4 (1.8)	21 (5.4)	50 (8.3)	19 (5.9)	6 (6.8)	
No change	600 (36.8)	51 (22.9)	91 (23.3)	225 (37.3)	175 (54.0)	58 (65.9)	

p-Value calculated based on ANOVA test/Chi-2.

### Health related characteristics of postpartum women

The health characteristics of the study participants are summarized in Table 2. The mean (SD) pre-pregnancy BMI was 26.3 (5.1) kg/m^2^. Lebanon had the lowest mean (SD) BMI of 25.8 (6.9) kg/m^2^, and Bahrain had the highest mean (SD) BMI of 28.2 (6.9) kg/m^2^ (*p*-value < 0.001). The majority of the study participants had a normal BMI (43%), 36.2% were overweight, 19.3% were obese, and only 1.6% were underweight. The highest proportion of normal BMI was in Lebanon (46.5%), and Jordan had the highest proportion of overweight (41.7%), Bahrain had the highest proportion of obesity among all, with 19.4, 13.4, and 4.5% in class I, II, and III respectively, while Saudi Arabia had the highest proportion of underweight (2.5%; *p*-value < 0.001). Only a small proportion of the respondents were diagnosed with COVID-19 (6.6%), with Bahrain having the highest percentage (20.5%), and Palestine had the lowest percentage (1.3%; *p*-value < 0.001). The vast majority of the study participants reported having health complications, with Palestine having the highest proportion (78.2%), and Jordan having the least (68.7%). Most of the participants reported having no diabetes (95.7%), gestational diabetes (93%), hypertension (98.6%), or thyroid disorders (96.4%). However, a large proportion reported mild to severe depression (87.4%). Mild depression was reported by 42.1% of the participants, with more than half of them being in Bahrain (51.4%), while 25.8% reported moderate depression, with the highest proportion being in Palestine (32%). Moreover, around 67% were anxious, with the highest proportion of mild anxiety being in Jordan (58.8%), moderate anxiety being in Palestine (18.6%), and severe anxiety being in Lebanon (5.1%). Most of the respondents were physically active (64%), with Jordan placing first (85.7%), and Bahrain placing last (43.9%; *p*-value < 0.001). Smoking during pregnancy was not high among the respondents. 22.7% reported smoking during pregnancy, with Lebanon having the highest proportion (33.4%) and Saudi Arabia having the lowest proportion (8%; *p*-value < 0.001).

**Table 2:** Health related characteristics of the study postpartum women (*n* = 1,630).

**Variable**	**All** ***n* = 1,630**	**Jordan** ***n* = 223**	**Palestine** ***n* = 391**	**Lebanon** ***n* = 604**	**Saudi** **Arabia *n* = 324**	**Bahrain** ***n* = 88**	***p*-Value**
**Mean (SD)**							
**Pre-pregnancy BMI kg/m** ^**2**^	26.3 (5.1)	26.8 (3.6)	26.2 (4.2)	25.8 (4.6)	26.8 (6.9)	28.2 (6.9)	<0.001
***n*** **(%)**							
**Body mass index (BMI)**							
Normal	580 (43.0)	72 (37.5)	137 (42.0)	226 (46.5)	120 (43.2)	25 (37.3)	<0.001
Thin	21 (1.6)	0 (0.0)	3 (0.9)	10 (2.1)	7 (2.5)	1 (1.5)	
Overweight	489 (36.2)	80 (41.7)	133 (40.8)	167 (34.4)	93 (33.5)	16 (23.9)	
Obese class I	190 (14.1)	37 (19.3)	37 (11.3)	72 (14.8)	31 (11.2)	13 (19.4)	
Obese class II	56 (4.2)	3 (1.6)	16 (4.9)	9 (1.9)	19 (6.8)	9 (13.4)	
Obese class III	13 (1.0)	0 (0.0)	0 (0.0)	2 (0.4)	8 (2.9)	3 (4.5)	
**Diagnosed with COVID-19**							
No	1,523 (93.4)	201 (90.1)	386 (98.7)	570 (94.4)	296 (91.4)	70 (79.5)	<0.001
Yes	107 (6.6)	22 (9.9)	5 (1.3)	34 (5.6)	28 (8.6)	18 (20.5)	
**Health problems**							
No	342 (25.1)	61 (31.3)	72 (21.8)	110 (22.3)	78 (28.1)	21 (30.9)	0.034
Yes	1,022 (74.9)	134 (68.7)	258 (78.2)	383 (77.7)	200 (71.9)	47 (69.1)	
**Diabetes**							
No	22 (95.7)	–	22 (95.7)	–	–	–	
Yes	1 (4.3)	–	1 (4.3)	–	–	–	
**Gestational diabetes**							
No	1,516 (93.0)	188 (84.3)	376 (96.2)	578 (95.7)	295 (91.0)	79 (89.8)	<0.001
Yes	114 (7.0)	35 (15.7)	15 (3.8)	26 (4.3)	29 (9.0)	9 (10.2)	
**Hypertension**							
No	1,607 (98.6)	220 (98.7)	382 (97.7)	598 (99.0)	321 (99.1)	86 (97.7)	0.404
Yes	23 (1.4)	3 (1.3)	9 (2.3)	6 (1.0)	3 (0.9)	2 (2.3)	
**Thyroid Disorders**							
No	1,572 (96.4)	217 (97.3)	368 (91.4)	587 (97.2)	315 (97.2)	85 (96.6)	0.085
Yes	58 (3.6)	6 (2.7)	23 (5.9)	17 (2.8)	9 (2.8)	3 (3.4)	
**Depression**							
No depression	93 (12.6)	16 (9.7)	14 (8.0)	35 (13.9)	23 (20.9)	5 (14.3)	0.004
Mild	310 (42.1)	76 (46.1)	61 (34.9)	106 (42.1)	49 (44.5)	18 (51.4)	
Moderate	190 (25.8)	42 (25.5)	56 (32.0)	63 (25.0)	23 (20.9)	6 (17.1)	
Moderately severe	85 (11.5)	23 (13.9)	30 (17.1)	20 (7.9)	9 (8.2)	3 (8.6)	
Severe	59 (8.0)	8 (4.8)	14 (8.0)	28 (11.1)	6 (5.5)	3 (8.6)	
**Anxiety**							
No anxiety	243 (32.8)	36 (21.8)	55 (31.1)	91 (36.0)	43 (39.1)	18 (51.4)	0.006
Mild	372 (50.3)	97 (58.8)	85 (48.0)	123 (48.6)	52 (47.3)	15 (42.9)	
Moderate	98 (13.2)	26 (15.8)	33 (18.6)	26 (10.3)	12 (10.9)	1 (2.9)	
Severe anxiety	27 (3.6)	6 (3.6)	4 (2.3)	13 (5.1)	3 (2.7)	1 (2.9)	
**Physical activity**							
Inactive	315 (36.0)	25 (14.3)	95 (41.9)	112 (37.6)	60 (44.4)	23 (56.1)	<0.001
Active	561 (64.0)	150 (85.7)	132 (58.1)	186 (62.4)	75 (55.6)	18 (43.9)	
**Smoking during pregnancy**							
No	796 (77.3)	49 (70.0)	211 (80.2)	275 (66.6)	207 (92.0)	54 (91.5)	<0.001
Yes	234 (22.7)	21 (30.0)	52 (19.8)	138 (33.4)	18 (8.0)	5 (8.5)	

### Daily intake of the main food groups according to usda

Daily consumption of the main food groups before and during COVID-19 pandemic is presented in Table 3. There was a substantial increase in the mean (SD) consumption of all the food groups, including bread, rice, and other cereals, fruits, vegetables, milk and milk products, white and red meat, and nuts (*p*-value < 0.05). Prior to the pandemic, Bahrain had the highest consumption of bread, rice, and other cereals (62.5%; ≥6 servings/day), fruits (55.6%; ≥2 servings/day), and vegetables (36.4%; ≥2.5 servings/day) compared to Jordan, Palestine, Lebanon, and Saudi Arabia. However, this difference was only significant in the bread, rice, and cereal group (*p*-value < 0.05). Saudi Arabia had the highest consumption of milk and milk products (20.5%; ≥3 servings/day), while Jordan had the highest consumption of white and red meats and nuts (26.7%; ≥5.5 servings/day). However, this difference was not significant. In contrast, Jordan had lower consumption of bread, rice, and cereals (25.6%; ≥6 servings/day; *p*-value < 0.05). After the COVID-19 pandemic, Bahrain had the highest consumption of bread, rice, and cereals, while Jordan had the highest consumption of fruits (66.7%; ≥2 servings/day) and a significant increase in consumption of vegetables (48%; ≥2.5 servings/day; *p*-value = 0.006). Other than that, Jordan had a significant increase in white and red meat and nut consumption and was the highest among the other countries even after the pandemic (51.3%; ≥5.5 servings/day; *p*-value < 0.001).

**Table 3:** Daily intake of the main food groups per country according to USDA.

**Food group**		**Number of servings before the pandemic** ***n*** **(%)**							**Number of servings during the pandemic** **n (%)**					***p*-Value**	***p*-Value** **# (Before vs. during the pandemic)**
	**All** ***n* = 1,630**	**Jordan** ***n* = 223**	**Palestine** ***n* = 391**	**Lebanon** ***n* = 604**	**Saudi** **Arabia** ***n* = 324**	**Bahrain** ***n* = 88**	***p*-Value**	**All** ***n* = 1,630**	**Jordan** ***n* = 223**	**Palestine** ***n* = 391**	**Lebanon** ***n* = 604**	**Saudi** **Arabia** ***n* = 324**	**Bahrain** ***n* = 88**		
**Bread, rice and other cereals** [**Mean (SD)**]	4.6 (3.2)	4.2 (2.4)	4.7 (3.9)	4.4 (2.5)	4.8 (3.6)	5.8 (2.9)	0.203	5.2 (3.8)	5.8 (3.8)	5.3 (4.6)	4.9 (3.0)	5.3 (3.9)	5.3 (2.9)		<0.001
**Bread, rice and other cereals** ***n*** **(%)**															
<6	320 (65.0)	58 (74.4)	110 (67.5)	95 (63.3)	48 (62.3)	9 (37.5)	0.018	265 (57.0)	25 (46.3)	98 (60.9)	84 (57.1)	48 (60.8)	10 (41.7)	0.18	0.003
≥6	172 (35.0)	20 (25.6)	53 (32.5)	55 (36.7)	29 (37.7)	15 (62.5)		200 (43.0)	29 (53.7)	63 (39.1)	63 (42.9)	31 (39.2)	14 (58.3)		
**Fruits** [**Mean (SD)**]	2.0 (1.6)	1.7 (1.1)	2.1 (1.6)	2.1 (1.8)	1.8 (1.4)	2.4 (2.6)	0.217	2.2 (1.6)	2.6 (1.7)	2.1 (1.5)	2.2 (1.6)	2.0 (1.7)	2.1 (1.5)	0.218	0.016
**Fruits** ***n*** **(%)**															
<2	231 (52.4)	54 (61.4)	68 (48.9)	59 (46.1)	42 (61.8)	8 (44.4)	0.078	183 (44.9)	21 (33.3)	64 (47.4)	54 (44.3)	38 (53.5)	6 (35.3)	0.162	0.078
≥2	210 (47.6)	34 (38.6)	71 (51.1)	69 (53.9)	26 (38.2)	10 (55.6)		225 (55.1)	42 (66.7)	71 (52.6)	68 (55.7)	33 (46.5)	11 (64.7)		
**Vegetables** [**Mean (SD)**]	1.9 (1.3)	1.8 (1.3)	2.0 (1.4)	1.8 (1.0)	1.7 (1.5)	2.5 (1.7)	0.055	2.3 (1.9)	2.8 (1.9)	2.2 (1.6)	1.9 (1.2)	2.4 (3.3)	2.3 (1.6)	0.045	<0.001
**Vegetables** ***n*** **(%)**															
<2.5	356 (76.7)	76 (77.6)	104 (72.7)	106 (80.9)	56 (80.0)	14 (63.6)	0.274	297 (67.8)	39 (52.0)	100 (71.4)	97 (75.2)	49 (68.1)	12 (54.5)	0.006	<0.001
≥2.5	108 (23.3)	22 (22.4)	39 (27.3)	25 (19.1)	14 (20.0)	8 (36.4)		141 (32.2)	36 (48.0)	40 (28.6)	32 (24.8)	23 (31.9)	10 (45.5)		
**Milk and milk products** [**Mean (SD)**]	1.8 (1.8)	1.7 (1.4)	1.8 (1.4)	1.9 (2.4)	2.0 (1.8)	2.1 (1.4)	0.768	2.0 (1.6)	2.3 (1.9)	1.9 (1.6)	1.8 (1.5)	2.1 (1.8)	2.0 (1.4)	0.321	0.015
**Milk and milk products** ***n*** **(%)**															
<3	378 (84.0)	80 (88.9)	111 (81.0)	113 (86.9)	58 (79.5)	16 (80.0)	0.32	323 (77.6)	47 (70.1)	105 (80.2)	100 (80.6)	56 (75.7)	15 (75.0)	0.473	0.001
≥3	72 (16.0)	10 (11.1)	26 (19.0)	17 (13.1)	15 (20.5)	4 (20.0)		93 (22.4)	20 (29.9)	26 (19.8)	24 (19.4)	18 (24.3)	5 (25.0)		
**White and red meats and nuts** [**Mean (SD)**]	3.7 (2.7)	3.7 (2.4)	3.5 (2.7)	3.6 (2.8)	3.8 (2.9)	3.8 (1.9)	0.939	4.2 (3.5)	5.4 (3.0)	3.7 (2.6)	3.6 (2.8)	4.1 (3.1)	6.7 (9.2)	<0.001	<0.001
**White and red meats and nuts** ***n*** **(%)**															
<5.5	328 (80.0)	77 (73.3)	96 (82.1)	84 (81.6)	58 (85.3)	13 (76.5)	0.319	263 (71.1)	37 (48.7)	86 (76.8)	76 (81.7)	54 (76.1)	10 (55.6)	<0.001	0.004
≥5.5	82 (20.0)	28 (26.7)	21 (17.9)	19 (18.4)	10 (14.7)	4 (23.5)		107 (28.9)	39 (51.3)	26 (23.2)	17 (18.3)	17 (23.9)	8 (44.4)		
**Adherence to food groups**	1.1 (1.4)	0.9 (1.1)	1.3 (1.5)	1.1 (1.3)	1.2 (1.4)	1.7 (1.5)	<0.001	1.4 (1.5)	1.5 (1.1)	1.4 (1.6)	1.3 (1.4)	1.5 (1.6)	2.0 (1.7)	<0.001	<0.001
**Adherence to food groups**															
**No/low (0–2)** ***n*** **(%)**	478 (84.6)	115 (89.1)	138 (82.6)	138 (84.1)	67 (82.7)	20 (83.3)	0.589	425 (78.4)	90 (80.4)	127 (77.9)	129 (80.6)	65 (78.3)	14 (58.3)	0.168	<0.001
**Moderate/high (3–5) n (%)**	87 (15.4)	14 (10.9)	29 (17.4)	26 (15.9)	14 (17.3)	4 (16.7)		117 (21.6)	22 (19.6)	36 (22.1)	31 (19.4)	18 (21.7)	10 (41.7)		
**Adherence to food groups**	**Before pandemic** ***n*** **(%)**							**During pandemic** ***n*** **(%)**							
**No/low (0–2)**	478 (84.6)							425 (78.4)							
**Moderate/high (3–5)**	87 (15.4)							117 (21.6)							

Moreover, there was a significant change in the adherence to food groups as per the USDA guidelines, which is presented in Table 3. Before the pandemic, around 84% of participants reported no/low adherence (0–2) to USDA guidelines, whereas only 15% reported moderate or high adherence (3–5) to the guidelines. On the other hand, during the pandemic, there was an increase in the proportion of subjects reporting moderate/high adherence (22%; *p*-value < 0.001).

Table 4 shows a clear presentation of the adherence to recommendations of USDA guidelines before and after the pandemic. Adherence to consumption of bread, rice, and cereals decreased during the pandemic to 29%, whereas adherence to the rest of the food groups (fruits, vegetables, milk and milk products, white and red meat, and nuts) significantly increased (*p*-value < 0.001)

**Table 4:** Adherence to recommended number of food groups according to USDA.

**Number of food groups**	**Before pandemic** ***n* (%)**	**During pandemic** ***n* (%)**	***p*-Value**
**0**	237 (41.9)	185 (34.1)	<0.001
**1**	169 (29.9)	157 (29.0)	
**2**	72 (12.7)	83 (15.3)	
**3**	40 (7.1)	56 (10.3)	
**4**	24 (4.2)	30 (5.5)	
**5**	23 (4.1)	31 (5.7)	

**Figure 1:**
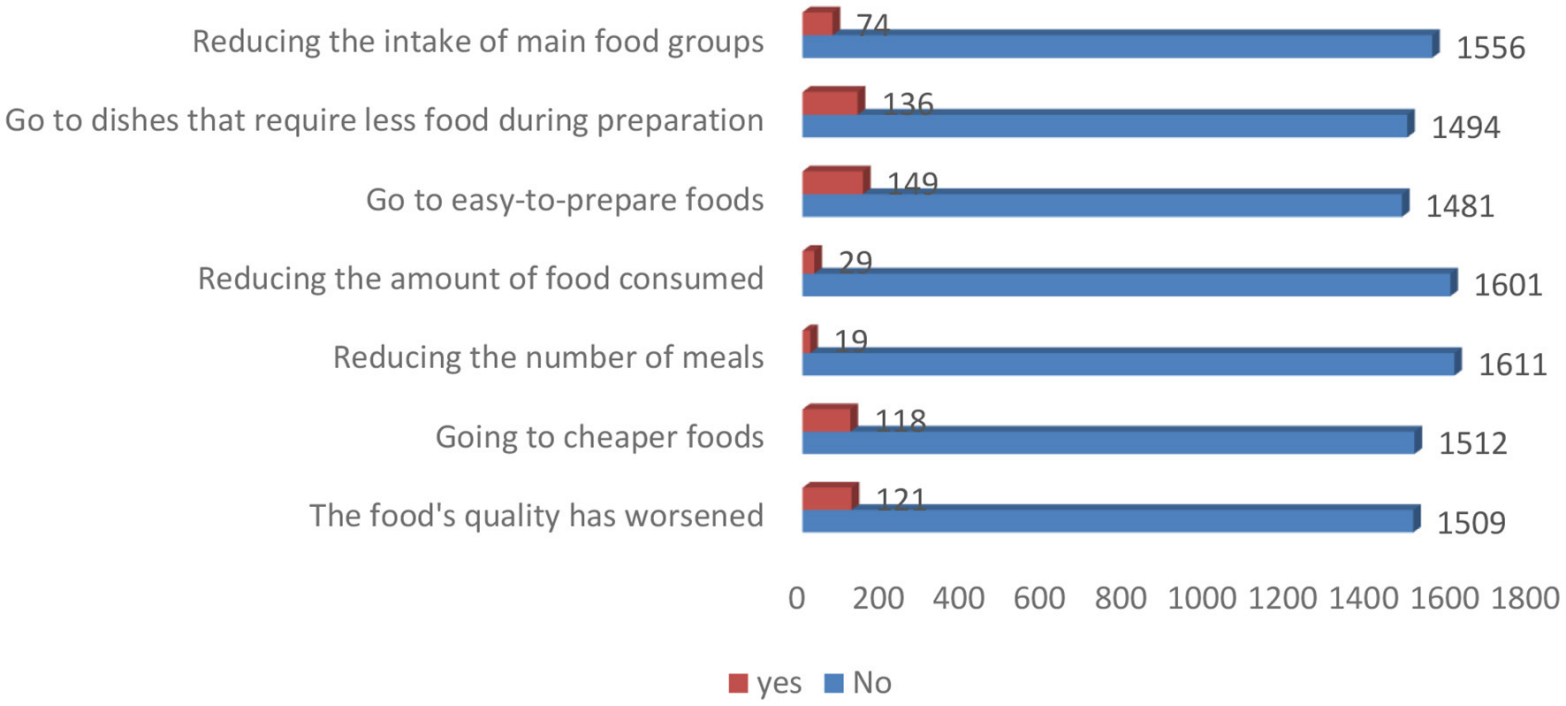
Mitigation measures adopted by post-partum women to cope with the situation during the pandemic.

Mitigation measures adopted by post-partum women to cope with the situation during the COVID-19 pandemic are presented in Figure 1. Majority of the women reported no change in food related behaviors during the pandemic. However, 9% stated that they go for easy to prepare foods, 8% go for dishes that require less food during preparation, and 7% reported worsened food quality and choosing cheaper food.

## Discussion

This study mainly focuses on comparing postpartum women's dietary intake of the main food groups and their adherence to the USDA dietary guidelines in five Eastern Mediterranean countries (Jordan, Palestine, Lebanon, Saudi Arabia, and Bahrain) before and during the COVID-19 pandemic. Overall, the study finds a decent improvement in the number of servings of all food groups and a slight improvement in their adherence to USDA dietary recommendations during the COVID-19 pandemic. Yet, the dietary consumption of the five food groups among the majority of the study participants was still below the recommendations.

The sociodemographic characteristics (age, education level, employment status, and family income) of postpartum women showed statistically significant differences between the five countries. The majority (80%) of the participants included were young adults (>25 years old), and more than half of the participants were educated and held a bachelor's degree, which is similar to most studies conducted on postpartum women (3, 9, 19). On the contrary, in a study conducted in Egypt, only a quarter of the postpartum women had a university education (2). In addition, more than 50% of the participants were unemployed and reported a decrease in their family income during the COVID-19 pandemic. In the Australian first-time mothers' study, only (1.8%) were unemployed and (88.6%) were keeping house and raising children (3). Although they were doing a full-time job, this group was considered unemployed in our study. Additionally, a decrease in family income was reported in 38 countries worldwide during the COVID-19 pandemic (20) as in our reported results.

Prior to pregnancy, the mean body mass index (BMI) of the participants was 26.3 (5.1) indicating that they were overweight. However, during postpartum (36.2%) were overweight, which might be related to breastfeeding or due to the reduction in their family's income during the pandemic. Moreover, a statistically significant difference between the BMI categories among the five countries was found. Previous studies conducted in China (9) and Australia (3) showed a lower percentage (28.1%) of overweight postpartum women, where the mean postpartum BMI was 26.2 (5.0) which is similar to our participants' pre-pregnancy BMI. Regarding the other health characteristics, most of the postpartum women were not diagnosed with COVID-19, diabetes or gestational diabetes, hypertension, and thyroid disorders, despite that (75%) reported having health problems. Other than that, 64% of our participants were physically active and 77% did not smoke during pregnancy, which decreased the risk of chronic diseases as mentioned above. Around half of them reported suffering from mild depression and anxiety, which is higher than the proportion reported in the Urban China study (9). These results might be related to the lower intake of fish or omega-3 polyunsaturated fatty acids during pregnancy and the postpartum period, as reported by Hamazaki et al. (21).

The COVID-19 pandemic and its socio-economic effects are likely to negatively impact the dietary intake and nutrition status of women, particularly during pregnancy and breastfeeding. Postpartum is a nutritionally vulnerable period since the maternal nutrient needs increase to meet physiological requirements, support the infants' and children's growth and development, and protect the health of the mother while breastfeeding. In the context of COVID-19, women may face additional risks affecting their diet quality and quantity (6). Unfortunately, the current literature lacks research articles that study the change in dietary intake from the main food groups and the adherence to the dietary guidelines and recommendations during the COVID-19 pandemic among postpartum women. However, there were a few longitudinal studies that examined the changes in the dietary intake of pregnant mothers in the third trimester and the first 40 days or months during the postpartum period, and a second study that examined postpartum maternal diet quality overtime in a population at high risk for obesity (10, 22, 23).

In general, our study results showed significant differences in the mean number of servings of bread, rice, and other cereals, fruits, vegetables, milk and milk products, and white and red meat and nuts consumption during the pandemic compared to before the pandemic. This was expected since more than 95% of the postpartum women did not reduce their intake of the main five groups, the number of meals, or the amount of food consumed to cope with the situation during the pandemic. Additionally, the only significant difference was in bread, rice, and other cereal consumption among the five countries before the pandemic, where 63% of Bahraini women had an intake of >6 servings. Even though during the pandemic, a difference was noticed in the consumption of vegetables, white and red meat and nuts, where 48% of Jordanian women consumed >2.5 servings of vegetables and 82% of Lebanese women consumed <5.5 servings of meat and nuts group. Moreover, there was no difference in the postpartum women's adherence to the five food groups' consumption before or during the pandemic when compared to the USDA's recommendations. Although, a statistically significant difference was detected in the moderate/high adherence when compared to the overall adherence (represented by an increase from 15 to 22% during the pandemic), the majority of the women showed low adherence.

Kay et al. (22) conducted a longitudinal observational study on a cohort of low-income, non-Hispanic black mothers in the postpartum period from 3 to 18 months to investigate the consumption of key food groups and the adherence to the 2010 Dietary Guidelines for Americans (DGAs). They found most mothers' median intake of grains was near recommended levels, but whole grain intake was low, suggesting most grains are coming from refined, processed foods. Another recent study reported a decrease in the Health Eating Index scores based on the number of servings consumed after 6 months postpartum in total grain products and whole grains compared to 3 months postpartum (23). The mean dietary grain intakes among two groups (community dwelling and residents in the Maternity Care Center) of lactating women in Shanghai during the puerperium were around the recommended values based on the Food Graph Reference for Retrospective Dietary Survey, although in the community sample it was slightly lower (24). Also, a study conducted on 32 healthy German women at 6 weeks post-partum revealed a decrease in carbohydrate intake (25). In this study, only 43% of women during the pandemic met the recommendations (>6 servings/day) for bread, rice, and other cereals. The mean dietary intake from this food group was below the recommendation. During the pandemic, a slight increase in the mean 5.2 (3.8) was noticed compared to the mean intake 4.6 (3.2) before the pandemic in the five countries.

Recently, Pligt et al. (3) used the Cancer Council of Australia's Food Frequency Questionnaire to assess the fruit and vegetable intake (servings/day) of postpartum women. Their results showed that approximately half of the participants (55.4%) met the fruit recommendations defined as two or more servings/day. Furthermore, no change was recorded in the median daily servings of fruits (two servings) at 3, 6, 9, 12, and 18 months during the periodical postpartum follow-up (22). Our results are in-line with the previous results where 55.1% of mothers consumed >2 servings of fruits during the pandemic and the mean number of servings significantly increased during the pandemic to reach 2.2 servings.

In the Shanghai study, all lactating mothers' mean intake of vegetables, tubers, and fruit was lower than the recommendations (24). In a large prospective cohort study conducted to explore the changes in vegetable intake between pregnancy and the postnatal period using the diet history questionnaire, researchers found that 44% of women were not meeting vegetable recommendations during pregnancy or in the postpartum period. Remarkably, at 4 months postpartum, 15% of women consumed the recommended number of servings of vegetables during pregnancy but did not meet the recommendation (26). Moreover, Pliget et al. (3) reported that 8.6% of all Australian postpartum women met the recommendations for vegetable intake, defined as >5 servings per day, with 44.9% of women consuming <2 servings per day of vegetables (27) and only 7.2% of total women met the recommendations for both fruit and vegetable intake (3). In our study, 32% of women met the recommendations (>2.5 servings/day) of vegetable intake during the pandemic, and the mean servings 2.3 (1.9) of vegetable intake was lower than the recommended amounts, although there was a significant increase in the mean vegetable serving during the pandemic among the five countries. Nevertheless, 68% of participants consume <2.5 servings per day. Our results agree with previous studies regarding the adherence to the dietary intake of vegetables but also disagree with other results due to not considering the women's dietary intake during pregnancy in the current study and the difference in the dietary guidelines used in each study

The postpartum women's milk and milk product intake was below the recommendations, and the mean intake was 2.0 (1.6) cups during the pandemic, which is higher than their intake 1.8 (1.8) before the pandemic. More than three-fourths (78%) of them consumed <3 cups daily during the pandemic. Kay et al. (22) assessed the dietary intake of postpartum women at each home visit using a computerized 24-h recall and investigated the adherence to the Dietary Guidelines for Americans. They found many mothers consuming dairy foods, but the median number of servings was approximately one-third of the recommendation of 3 servings per day. Our results disagree with Kay et al. because we have approximately one fourth of our participants (22%) consuming >3 servings per day. In agreement with our results, at 6 months postpartum, Cuervo et al. (28) observed a decrease in milk and milk product intake. Similar observations were reported at 26 weeks postpartum (29) and at 4 months postpartum in Australian women with obesity (30). Similarly, in the Shanghai study, the milk intake was low in the 2 groups of postpartum lactating mothers, with an overall mean intake of approximately a quarter of the recommended quantity (24).

The last food group studied was proteins, or white and red meats and nuts. Results showed that during the pandemic, 71% of the postpartum women had an intake that was below the recommendations. Their mean daily intake during the pandemic was 4.2 (3.5) ounces, while it was less before the pandemic 3.7 (2.7 ounce/day). Only 29% of the participants during the pandemic met the recommendation of > 5.5 ounces/day. On the contrary, the mean meat/poultry, egg, and fish/shrimp intakes of all studied Shanghai lactating mothers were higher than the recommendations (24). Furthermore, in the Kay et al. study, almost all mothers consumed protein foods (i.e. meat, poultry, seafood, eggs, soy products, nuts, and seeds) and they met the recommendations. Similar to our results, a study that followed mothers from pregnancy to 4 months postpartum reported a decrease in the quality and quantity of meat consumption (30). Research also focused on fish and omega-3 consumption postpartum, and they linked the higher intake with a reduction in postpartum depression risk at 6 months after delivery and serious mental illness at 1 year after delivery (21).

Due to differences in selected postpartum periods, the results of research articles on the dietary intake of postpartum women varied. For instance, one study looked at 26 weeks postpartum (29) and showed a decrease in the intake of fruits, vegetables, cereals, and oils. Another study looked at 6 months postpartum and found a decrease in the intake of cereals, vegetables, fruits, and dairy products (28). Moreover, a different study looked at 4 months postpartum and found a reduction in the consumption of milk, meat, and oil (30). Recently, in research that evaluated and compared the immediate postpartum (up to 40 days after delivery) dietary intake with the third trimester of pregnancy, a significant lower intake of fruits, vegetables, salted, and sweet cereals was observed, but there was no change regarding adherence to the Mediterranean diet (10). Lebrun et al. studied dietary intake and diet quality from the third trimester of pregnancy to 6 months postpartum using completed 2–3 Web-based 24-h recalls. They discovered that the total intake of several micronutrients (vitamins A, C, D, group B vitamins, iron, magnesium, zinc, calcium, phosphorus, manganese, and copper) and supplements intake decreased significantly over time, while the total Canadian healthy eating index score and its components did not change (23). Additionally, similar results were found regarding the dietary intake of postpartum women at different times in cross-sectional studies, where mothers were not meeting the recommended intake levels, especially fruits and vegetables, while they were consuming excessive amounts of sugar-sweetened beverages (3, 31–34).

Several studies (10, 22–24) found that postpartum women did not adhere to the recommended number of food groups. These results are similar to our findings, but the difference was that we studied the change in adherence during the COVID-19 pandemic according to USDA dietary guidelines. A statistically significant difference was found in women's adherence to the five-food groups, although the percentage of women adhering to the five food groups was 4.1% before the pandemic and 5.7% during the pandemic. This means most women have low adherence either before or during the pandemic. Unfortunately, no studies were found about postpartum women's dietary adherence to guidelines during the COVID-19 pandemic except through pregnancy (5, 35). For instance, in an internet-based cross-sectional survey, the adherence of pregnant Spanish women to the Mediterranean diet showed that more than half (57.8%) had moderate adherence, around a third (35.6%) had high adherence, and only a small percentage (6.6%) reported low adherence (5).

This study has several strengths. It is the first of its type in the Eastern Mediterranean Region that examined the change in postpartum women's dietary intake and adherence to USDA dietary guidelines before and during the COVID-19 pandemic. In addition, it included an acceptable number of participants from five different countries. However, the study has a number of limitations, including that it was an internet-based cross-sectional survey and that bias selection might have occurred. Besides the limits/cutoffs of the five food groups, dietary intake was adopted from the USDA dietary guidelines because of a lack of common Eastern Mediterranean guidelines, yet this may not give us the right picture about the Eastern Mediterranean women's dietary status. Lastly, this study contained many questions, which designed to identify the woman's dietary intake situation during the pandemic, thereby determining the amount of food consumed and identifying the nutrients level was not possible.

## Conclusions

The COVID-19 pandemic has positive effects on dietary intake and adherence to guidelines among postpartum Eastern Mediterranean women. However, a large proportion of our study participants maintained their dietary intake below the recommended amounts, and low adherence to the five food groups. Therefore, urgent actions should be taken to protect postpartum women and their children from the effects of low dietary intake, particularly during pandemics and lockdowns, through focusing on nutrition support programs, nutrition education, and the promotion of adopting healthy eating behaviors and lifestyle. Further research is needed to assess dietary intake in quality and quantity in a larger cohort of postpartum women using longitudinal follow-up studies and to identify mothers at higher risk of inadequate intake to cover them in nutrition intervention programs.

## Data availability statement

The raw data supporting the conclusions of this article will be provided by the corresponding authors when requested and without undue reservation.

## Ethics statement

The studies involving human participants were reviewed and approved by Ethics Committee in Scientific Research of University of Jordan (19/2020/585). The patients/participants provided their written informed consent to participate in this study.

## Author contributions

RT: conceptualization, data curation, formal analysis, investigation, methodology, project administration, supervision, validation, and writing—original draft preparation. NA-B: data curation, methodology, writing—original draft preparation, and writing—review and editing. NA-A: methodology, writing—original draft preparation, and writing—review and editing. HA: writing—original draft preparation and review and editing. RH: data curation, formal analysis, methodology, and writing—review and editing. RQ: conceptualization, data curation, formal analysis, and writing—review and editing. RS: data curation, methodology, and writing—review and editing. SA, JA, and KB: conceptualization, data curation, methodology, and writing—review and editing. AB: data curation and writing—review and editing. EB: conceptualization, data curation, and writing—review and editing. MH: conceptualization, data curation, formal analysis, investigation, methodology, project administration, supervision, validation, writing—original draft preparation. All authors have read and agreed to the published version of the manuscript.

## Research group

Bahrain (Mahmoud Samy Ismail and Nada Omar Abduljawad; King Hamad University Hospital); Lebanon (Mariane Abi Nasr, Sarah Obeid, Mohamad El Hajj: Lebanese University; Chadi Fakih: Al Hadi Laboratory and IVF Center); Palestine (Diala Abu Al Halawa: Al-Quds University; Firas Abdel Jawad: Makassed Hospital; Nabil Thawabteh: Makassed Hospital; Hazem Agha: Al Quds University); Kingdom of Saudi Arabia (Areej Alamer: Ministry of Health (MOH); Majid M Alkhalaf: National Nutrition Committee (NNC), Saudi Food and Drug Authority (Saudi FDA).

## Conflict of interest

The authors declare that the research was conducted in the absence of any commercial or financial relationships that could be construed as a potential conflict of interest.

## Publisher's note

All claims expressed in this article are solely those of the authors and do not necessarily represent those of their affiliated organizations, or those of the publisher, the editors and the reviewers. Any product that may be evaluated in this article, or claim that may be made by its manufacturer, is not guaranteed or endorsed by the publisher.

## Author disclaimer

The authors alone are responsible for the views expressed in this article and they do not necessarily represent the views, decisions or policies of WHO or the other institutions with which the authors are affiliated.
